# Study on knowledge and perceptions on the uptake of non-medicinal cannabis-substances and preparations by Portuguese consumers: Borderline issues

**DOI:** 10.1016/j.heliyon.2024.e40827

**Published:** 2024-11-29

**Authors:** Alexandre Elias, Catarina Rosado, Maria do Céu Costa

**Affiliations:** a CBIOS, Universidade Lusófona's Research Center for Biosciences & Health Technologies, Campo Grande 376, 1749-024, Lisboa, Portugal; b IPLUSO, ERISA-Escola Superior de Saúde Ribeiro Sanches, Rua do Telhal aos Olivais, 8-8^a^, 1900-693, Lisboa, Portugal

**Keywords:** *Cannabis sativa* L., Hemp, Food supplements, Consumer perception, Cannabinoids, CBD, THC

## Abstract

*Cannabis sativa* L.-based food supplement products in pharmacies and para pharmacies in Portugal increased by 84 % between 2021 and 2022, arousing consumers' curiosity. However, information about these products is limited, and consumers are not aware of the restrictions in current European regulations. This study aims to understand Portuguese consumers' perceptions of cannabis products and identify the distribution channels and market strategy. A cross-sectional investigation on the consumption of non-medicinal products derived from cannabis occurred using a survey that aimed to collect data covering four main research questions: consumer information, consumed products, level of satisfaction, and used channels for purchasing products. Applying an original questionnaire aimed at the public via email and social networks, 176 responses were collected, where a high degree of satisfaction with taking cannabis-based products was evident, with sleep disorders and the promotion of well-being as the reasons (48,5 %) that led to the majority of respondents to seek out these products. Health professionals are already recommending cannabis-derived products; however, most respondents are unable to differentiate a food supplement from a medicine. Online purchase was the respondents' favourite choice, and respondents (93 %) were unaware of the properties of food supplements in general. Consumers ignore that the parts of the cannabis plant, whose active ingredients they expect to have a greater capacity to promote well-being, namely cannabinoids, are not authorized by the European Food Safety Authority (EFSA) to be marketed in foods or dietary supplements.

Results also show that the influence of media in Portugal is significant in the choice of products, together with the lack of information on cannabis-based supplements and medicines, highlighting the need for a pro-consumer review, and promoting conscious and informed choices. Thus, we propose creating a Community Knowledge on Food Supplements linking academics, stakeholders, and authorities.

## 1 Introduction

The historical roots of cannabis use can be traced back to ancient China, where it found application in religious ceremonies, for spiritual and medicinal purposes to prevent and treat diseases due to its psychotropic effects [[1], [2], [3], [4]]. The earliest records of therapeutic use date back to Chinese medicine around 2300 BC. In India, cannabis became integrated into the Hindu religion and was later presented to the European continent during the period between 1000 and 2000 B.C. Later, the plant arrived in the Americas, first in Chile, and in 1606 it began to be cultivated in North America [5]. Western medicine began to explore the properties of cannabis at the beginning of the 19th century, expanding its use in the 20th century. However, the plant faced marginalization due to prejudice and misinformation, impeding research into its medicinal benefits [2,6] (see Fig. 3, Fig. 4, Fig. 5, Fig. 6, Fig. 7, Fig. 8, Fig. 9, Fig. 10, Fig. 11).

Cannabis, one of the oldest cultivated plants, offers remarkable nutritional and medicinal benefits [7,8]. It is a versatile plant, and its fiber can be used for industrial purposes, seeds for food, and flowers for medicinal purposes [7,9,10]. Raw, cooked, or pressed cannabis seeds are rich sources of fiber, proteins, and fats with high nutritional value [3,8]. According to a report by the UN's International Narcotics Control Board [11], the UK produced almost half of the world's legal cannabis in 2016 at a whopping 95 tons of production, followed by Canada's 80.7 tons of production, with Portugal and Israel trailing at 21 tons and 9.2 tons respectively.

In the last decade, there has been a notable increased interest in cannabis, covering medicines, food supplements, benefit-risk studies, regulation, and other topics [12,13].

**Fig. 1:**
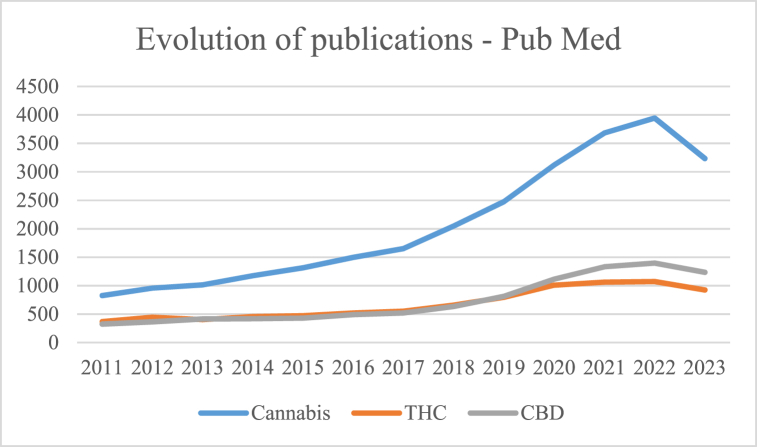
Evolution of publications on “Cannabis”, “THC” and “CBD” - 2011–2023 (Pub Med).

A bibliographic analysis in PubMed of the last 10 years, using the keywords "Cannabis", "THC" (Tetrahydrocannabinol), and "CBD" (Cannabidiol), reveals a marked growth in the number of publications related to these terms, as shown in Fig. 1

**Fig. 2:**
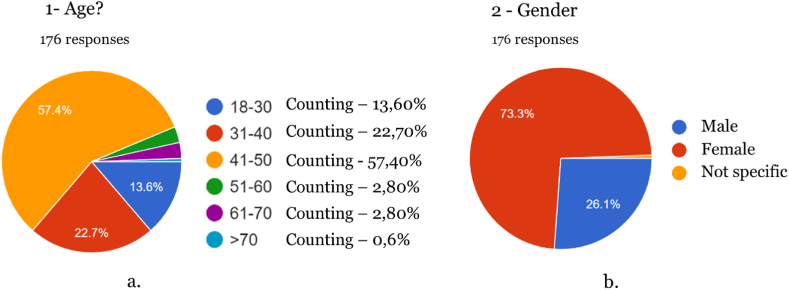
A- Age of respondents; b- Gender of respondents.

**Fig. 3:**
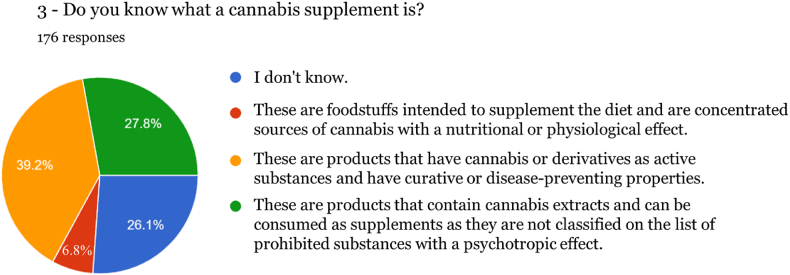
Knowledge about Cannabis-based supplements.

**Fig. 4:**
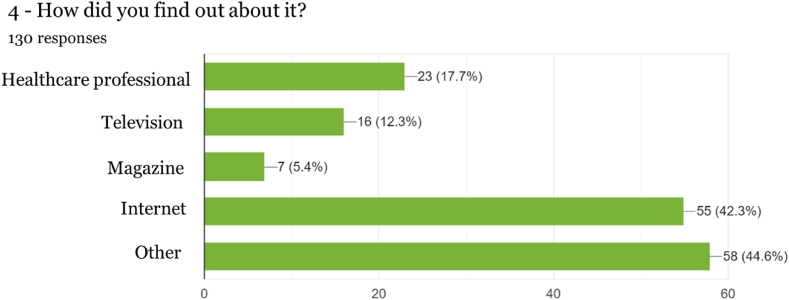
Information pathways.

**Fig. 5:**
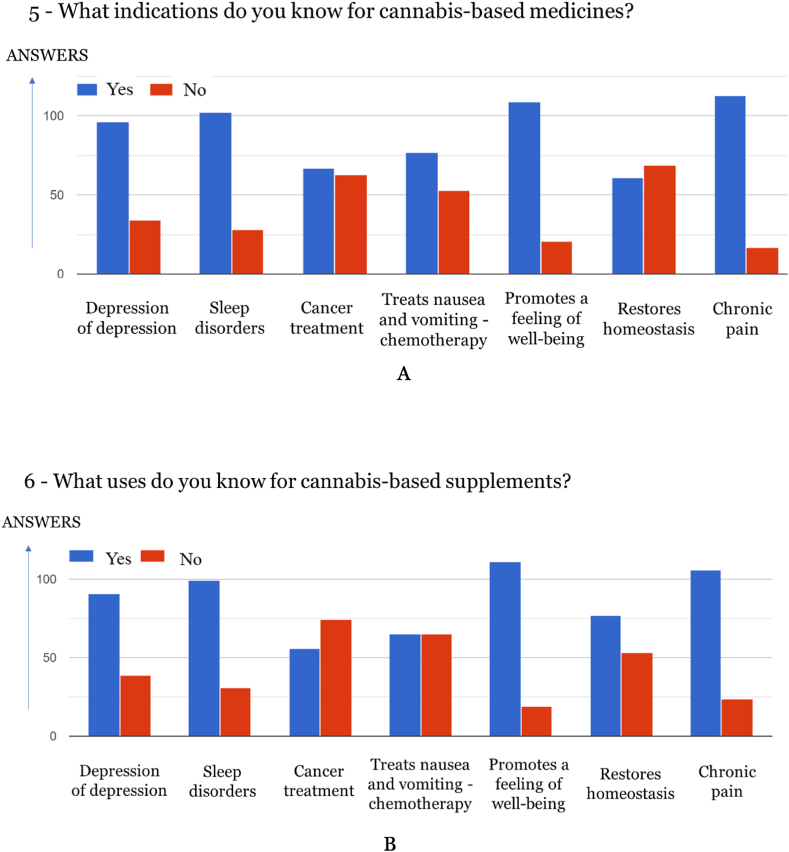
*A* Indications for cannabis-based medicines (130 responses, yy axis) B Uses of Cannabis-based supplements (130 Responses, yy axis).

**Fig. 6:**
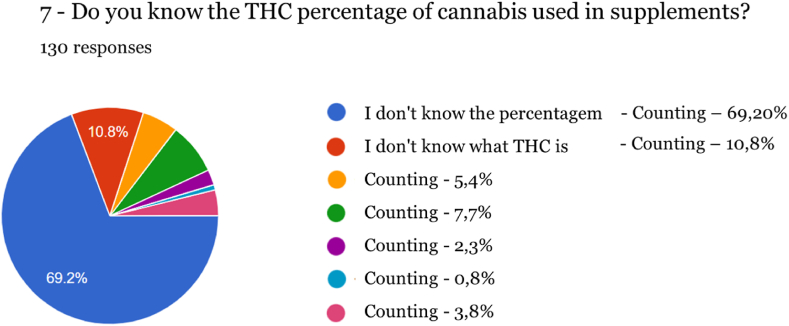
Percentage of THC in supplements.

**Fig. 7:**
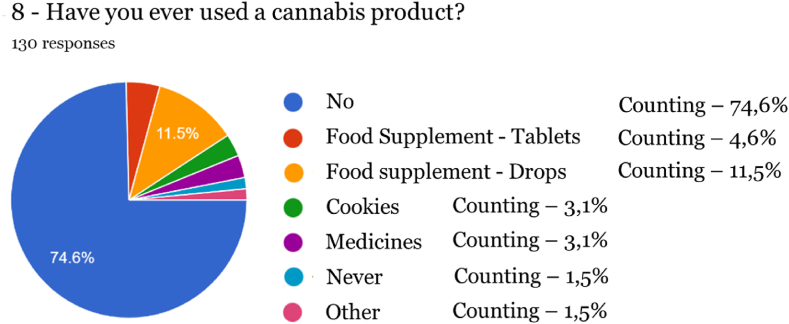
Adherence to Cannabis-derived products.

**Fig. 8:**
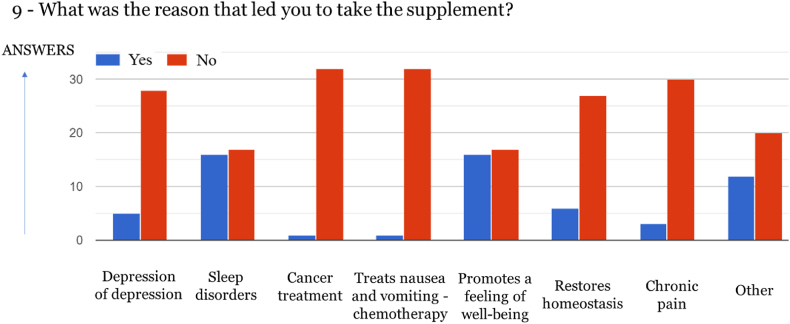
Reason for taking the supplement (33 answers, yy axis).

**Fig. 9:**
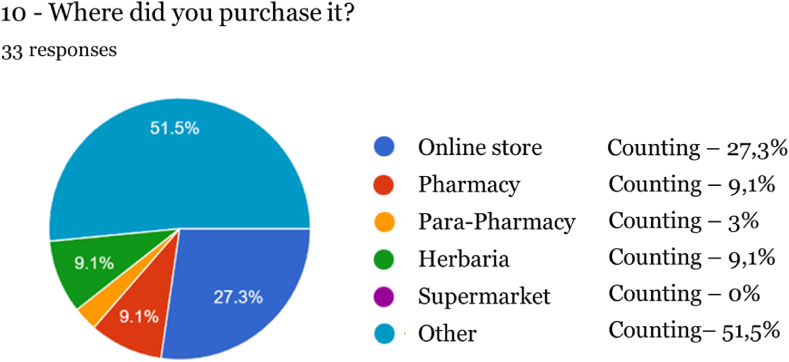
Acquisition location.

**Fig. 10:**
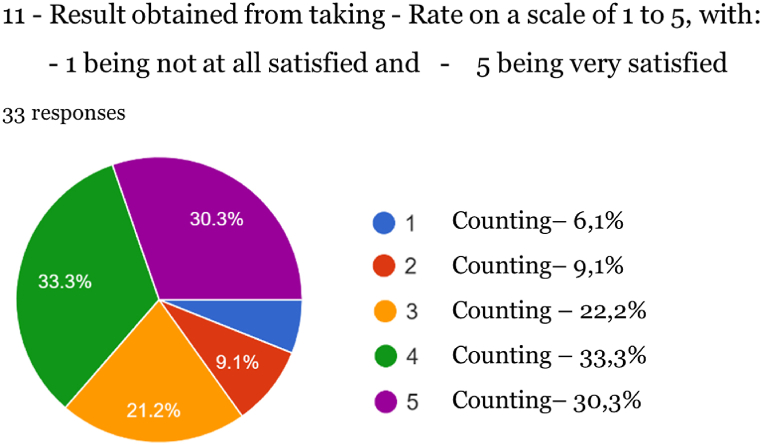
Satisfaction with taking CBD supplements.

**Fig. 11:**
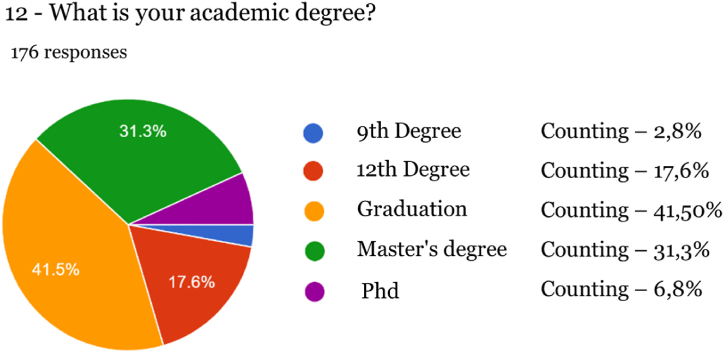
Academic degree of respondents.

Although the number of scientific publications in the literature on the subject is growing, the dissemination of information to consumers does not seem to show the same trend.

Regarding the marketing of these products in Portuguese pharmacies and para pharmacies, an increase of 84 % in 2022 compared to the previous year, as can be seen in Table 1, is probably attributable to a rise in consumers' interest in this type of product as well as novel products in the market in general.

**Table 1:** Sales of products based on *Cannabis sativa* L., THC and CBD in Portugal, 2021-22.

Channel \ Molecule	MAT May 2021 Sales Un	MAT May 2021 Sales Eur SP	MAT May 2022 Sales Un	MAT May 2022 Sales Eur SP
**ParaPharmacy**	2616	88 165,00	4600	122 762,36
CANNABIDIOL	929	14 757,21	2384	44 292,25
*Cannabis sativa*	1687	73 407,79	2379	85 855,39
**Pharmacy**	15 102	441 286,44	27 981	751 906,57
CANNABIDIOL	10 755	300 598,26	20 379	484 507,10
*Cannabis sativa*	4300	133 647,43	7242	193 948,44
TETRAHYDROCANNABINOL	363	157 346,59	888	259 646,30
Grand Total	17 718	529 451,44	32 581	874 668,93

Un = 1 Unit (1 Pack); Eur SP = appreciation of units sold at Street Price.

### 1.1 Background

Trends to the decriminalization of the use of cannabis in some countries, on the one hand, and the free use of cannabis non-psychotropic products such as cookies, candies, cakes, and food supplements, on the other hand, have met the requests of a market that is always looking for new things. (see Fig. 2)

Different varieties of *Cannabis sativa* L. were developed worldwide, some for medicinal use, high-CBD or high-THC, or a mixture of CBD and THC, and others with lower levels of CBD and trace THC (<0.3 %) authorized by the authorities for purposes other than medicinal, including human and animal food.

The use of cannabis in Portugal is currently decriminalized, as a result of the decriminalization of all drugs in Portugal in 2001. Under decriminalization, both production and sale remain illegal, but law enforcement does not prosecute individuals for the possession of small amounts of cannabis. Legalization, however, allows more stringent regulation and taxation. Furthermore, the medical use of cannabis was legalized in 2018. In this context, although there are directives from the Council and the European Parliament, and a legal framework in Portugal, there are still some flaws in their provision for Cannabis-based products as foods. Any extract from the *Cannabis sativa* L. plant and derived products containing cannabinoids, namely tetrahydrocannabinol (THC) and cannabidiol (CBD), is considered medicinal cannabis, to be authorized by Health Authorities according to the EU Code of Medicines or under a specific legal framework [14,15]. Hemp seed oils and other derivatives have limited THC contamination defined by Commission Regulation (EU) 2023/915 to be used as food ingredients. Other cannabinoid products, which are not classified as psychotropic may be candidates to be classified as novel foods (NF), since no history of consumption has been demonstrated before May 15, 1997, and as such can only be placed in the market after safety evaluation by the European Food Safety Authority (EFSA) under European Union (EU) Regulation 2283/2015 [16]. This is the case of Cannabidiol (CBD). n 2022 EFSA issued a statement [17], summarising the state of knowledge on the safety of CBD consumption and highlighting areas where more data are needed. The identified significant uncertainties and data gaps, lead the Experts Panel to conclude that the safety of CBD as a NF cannot currently be established.

Worthy of note, for Cosmetics, the substance cannabidiol (CBD), as a resin or cannabis preparation, is still included in Table I-C attached to the Portuguese Decree-Law no. 15/93, of January 22 to the applicable control measures to controlled substances therein. However, cannabidiol - derived from the extract or tincture or resin of cannabis, is according to the EU Cosmetics Regulation provision an identified ingredient that may have authorized functions as an antioxidant, anti-sebum, skin conditioning, and skin protecting. These different regulatory national frameworks for the constituents of cannabis, and in particular for Cannabidiol (CBD), are not communicated to consumers and raise some doubts among the general population that many products containing these ingredients are being marketed dubiously. The same questions arise with extracts that have become very popular [18]. In fact, In Portugal, the inclusion of CBD or other cannabinoids, which exist naturally in the cannabis plant, is not permitted, as they are obtained through the preparation of extracts or tinctures of Cannabis or its resin. The substances “cannabidiol - derived from extract or tincture or resin of cannabis” and “Cannabis sativa leaf extract” are designations appearing in the EU database CosIng - Cosmetics Ingredients but their inclusion in cosmetic products is not authorized in Portugal. However, the use of substances/preparations obtained from plant seeds with a THC content ≤0.2 %, such as cannabis seed oil, from varieties registered in the Common Catalog of Varieties of Agricultural Species, are exempt from this prohibition. Other sources of CBD are under analysis in the European Union and the World Health Organization. The use of these substances in cosmetics must be analyzed case by case and always requires a safety assessment.

### 1.2 Study context and local regulation issues

To date, no research has been conducted linking the use of cannabis, cannabinoids, and the main non-psychotropic component, cannabidiol (CBD), with national and European regulatory guidelines and public opinion on cannabis-derived products.

Portugal has a pioneering legal framework for the use of medicines, preparations, and substances based on the cannabis plant for medicinal purposes, namely their prescription and dispensing in a pharmacy. This legal framework aimed to make treatment with medicines, preparations, and substances based on the cannabis plant accessible, guaranteeing i) that the preparations made available meet all the requirements regarding the demonstration of their quality and safety, thus contributing to the safeguarding and protection of public health and ii) preventing the misuse of medicines, preparations, and substances based on the cannabis plant under the United Nations Convention against Illicit Traffic in Narcotic Drugs and Psychotropic Substances.

Thus, the entire production chain, from the cultivation of the plant to its preparation and distribution, is known and controlled, making it possible to guarantee that the products are produced per all good practices and applicable requirements. This ensures that patients have access to products that have demonstrated their quality and safety, not being exposed to unnecessary and avoidable risks, preventing their misuse, and limiting their use to cases where conventional treatments have not produced the expected effects or caused relevant adverse effects. The use of these products depends on the doctor's clinical assessment, considering the approved therapeutic indications. Dispensing of these products can only be carried out at the pharmacy upon presentation of a medical prescription. From a future perspective, it is estimated that in the short term, other EU Member States will adopt legislative measures identical to those in Portugal. Also, the planning of EMA's Community Herbal Monograph for Cannabis-Based Medicines in the medium term is under discussion.

The evolution of activities related to cannabis for medicinal purposes in Portugal can be illustrated by indicators of the number of entities with requests for final authorizations issued to carry out activities with cannabis for medicinal purposes in Portugal, namely cultivation, manufacturing, distribution, import and export of cannabis plants, preparations and substances based on the cannabis plant (Table 2).

**Table 2:** Number of authorizations issued by the Health Authority for cannabis-related activities for medicinal purposes (2017–2023).

Final Authorization Issued	2017	2018	2019	2020	2021	2022	2023 1S
Cultivation	2	3	5	9	18	20	21
Manufacturing				2	4	9	13
Importation/exportation	2	3	5	9	18	27	27
Commercialization				2	5	12	15

There are at present 8 products placed on the Portuguese market consisting of herbal substances and preparations based on the cannabis plant authorized for medicinal purposes, under a marketing authorization procedure designated as “ACM” (Table 3). A 9th medicinal product is Sativex, a mouth nebulizer that contains cannabinoid extracts from the Cannabis plant: each single 100-μL spray contains 2.7 mg of delta-9-tetrahydrocannabinol (THC) and 2.5 mg of cannabidiol (CBD).

**Table 3:** Medicinal products based on cannabis authorized by Portuguese Competent Authority for Medicines (Infarmed, I.P.).

Name of the Product	Active Substance(s)	Pharmaceutical Form
Satalliv	Canabidiol (CDB)	Oral solution, 10 mg/mL
Satalliv	Delta 9-tetrahidrocanabinol (THC)	Oral solution, 10 mg/mL
Satalliv	Delta-9-tetrahydrocannabinol (THC) + Cannabidiol (CBD), *Cannabis sativa* flower	Oral solution, 2 mg/mL THC + 10 mg/mL CBD
Satalliv	Delta-9-tetrahydrocannabinol (THC) + Cannabidiol (CBD), *Cannabis sativa* flower	Oral solution, 25 mg/mL THC + 25 mg/mlL CBD
Satalliv	Delta-9-tetrahydrocannabinol (THC) + Cannabidiol (CBD), *Cannabis sativa* flower	Oral solution 5 mg/mL THC+ 20 mg/mL CBD
Tilray Oral Solution THC 5 CBD 20	Delta-9-Tetrahydrocannabinol (THC PFV) + Cannabidiol (CBD PFV), Herbal preparation, *Cannabis sativa* extract	Oral Solution 5 mg/mL THC + 20 mg/mL CBD
Hexacan - Hexa 01 High-THC 20 %	Delta-9-tetrahydrocannabinol (THC) + Cannabidiol (CBD), *Cannabis sativa* Flower	Herbal Substance for inhalation by vaporization 20 % THC + ≤ 1.0 % CBD
Tilray Dry Flower THC 18	Delta-9-tetrahydrocannabinol (THC) + Cannabidiol (CBD), *Cannabis sativa* Flower	Herbal Substance for inhalation by vaporization 18 % THC+ ≤ 1.0 % CBD
Sativex	Delta-9-Tetrahydrocannabinol (THC PFV) + Cannabidiol (CBD PFV), Herbal Preparation, *Cannabis sativa* extract	Mouth spray solution 27 mg/mL THC + 25 mg/mL CBD
Epidyolex	Cannabidiol (CBD) Orphan drug	Oral solution 100 mg/mL CBD

Sativex is used in multiple sclerosis (MS) to improve symptoms related to muscle stiffness, also called “spasticity”, when there is an increase in “muscle tone”. Sativex is used when patients are refractory to conventional medications and have not achieved improvements to their stiffness/spasticity. The Marketing Authorization Holder is GW Pharma (International) B.V. The RRP is €475.27 with a 37 % contribution from the Portuguese government. Finally, Epidyolex (cannabidiol), in Portugal, is included in an early access program, approved for patients with Lennox-Gastaut or Dravet syndrome according to conditional rules in that program.

There are no authorized food supplements on the EU market claiming to contain CBD since, as noted earlier, their notification to the authorities in order to enter into the market as a potential novel food is subject to a safety assessment that has not yet been completed by EFSA.

Nevertheless, food supplements can be found in the market with hemp derivative ingredients under-regulated levels for use in foods. The Portuguese Food Authority (DGAV) is the competent entity for authorizing the cultivation of *Cannabis sativa* to obtain fibers and seeds not intended for sowing, including for food use or animal feed or the manufacture of food or compound feed for animals. In food, only the following ingredients obtained from cannabis have a history of consumption: seeds, seed oil, seed flour; and partially defatted seeds. Thus, flowers, leaves, and extracts from any part of the *Cannabis sativa* L. plant are unauthorized new foods and cannot be placed on the market as such, or even as food ingredients. They are, thus, either Novel Food or unauthorized foods obtained from *Cannabis sativa* L. extracts if containing cannabinoids, namely cannabidiol (CBD). THC in food is a contaminant according to EU Regulation No. 2023/915, with maximum limits of 3 mg/kg for seeds, partially defatted flour and seeds, and 7.5 mg/kg for oils.

Within this context, and given the complexities associated with the legal framework and the products in the market, this study aims to understand the perceptions of Portuguese consumers of cannabis products and identify their points of sale and market strategies.

## 2 Materials and methods

A cross-sectional study of the consumption of cannabis-based products was carried out using an original survey. The survey aimed to collect data covering four main research questions.

Q1 Are consumers properly informed about cannabis products?

Q2 What are the most consumed products?

Q3 How satisfied are consumers with the expected results?

Q4 Which channels are most used to purchase the products?

The survey was evaluated and validated beforehand by 10 health and nutrition experts, then conveyed to the general public, and disseminated by email and social networks between March and April 2022.

The questions presented in Table 4 were used as the main guide for this research.

**Table 4:** Questions prepared for carrying out research.

N.°	QUESTION
1	Age
2	Gender
3	Do you know what a cannabis food supplement is?
4	How did you find out about it?
5	What indications do you know for a cannabis-based medicine?
6	What uses do you know of for cannabis-based supplements?
7	Do you know the THC content of cannabis used in food supplements?
8	Have you ever used a cannabis product?
9	What prompted you to take the food supplement?
10	Where did you buy it?
11	Results obtained? Please rate on a scale of 1–5, with 1 being not at all satisfied and 5 being very satisfied.
12	What is your academic degree?

The survey included two knowledge assessment questions to determine whether the participants understood the definitions that characterize medicines and food supplements. This allowed for a more informed interpretation of the data collected. These questions were incorporated into questions 3 and 8, asking participants about their knowledge of cannabis-based supplements and whether they had ever consumed cannabis products. In the event of negative answers, the survey automatically moved on to the last stage, eliminating questions that were not pertinent in this case, such as details about indications for medication and the use of cannabis-based supplements, the source of knowledge about these products, and satisfaction with their consumption. 176 individuals participated in the survey aged between 18 and 70 (the most prevalent age interval between 41 and 50 years old). The data obtained via the Google Forms platform were exported to a table in Microsoft Excel software. This data was then transferred to SPSS 27.0 statistical software (IBM, NY, USA) for a univariate descriptive analysis, which included frequency measures. Spearman's correlation analysis was used to assess significant correlations between variables, with a statistical confidence level of 95 %.

The ethical principles for medical research involving humans as stated in the Declaration of Helsinki were followed. Consent from participants respected autonomy and ensured they were fully aware of their involvement was obtained before they took the survey. The purpose was explained, the type of questions they would encounter, and how their responses would be used under GDPR, the Data Protection Regulation in EU requirements. The operationalization of the survey was carried out through a questionnaire disseminated through social networks, with questions relating to Cannabis indications, sales channels, age, and gender, to build a pattern of users and also collect the level of information that the general public has about Cannabis-derived products. The results were analyzed without any identification of the participants. Only the Responsible Investigators had access to the data. After the end of the study, all data collected was kept on a computer protected by a password, for the period necessary for the analysis and writing of scientific works. This data will be kept for a period not exceeding 5 years, after which it will be destroyed.

## 3 Results

The survey aimed to collect informative data, such as consumers' perception of the accessibility and effects of Cannabis-products, most consumed products, level of satisfaction, and the sales channels used to obtain the products. However, some questions, such as gender and level of education, were introduced to explore potentially interesting correlations for the topic, regarding the characterization of the sample of respondents who consume these products.

A univariate descriptive analysis was carried out, with calculations of respective frequency measures to verify the existence of significant correlations. Due to the exploratory nature of the study, correlation analysis aimed to understand patterns and trends in the use of supplements, supported by factors. The correlation analysis was developed to identify these factors, namely knowledge in conjunction with behaviours.

The most significant correlation found was related to the issue of indications/uses of Cannabis-based supplements and medicines (Table 5).

**Table 5:** Correlation analysis data.

	F1		F2		F3		F4		F5		F6		F7	
	Rs	P	Rs	P	Rs	P	Rs	P	Rs	P	Rs	P	Rs	P
Q1	0.603	0.000	0.160	0.069	0.013	0.888	0.035	0.693	0.101	0.255	0.076	0.389	−0.012	0.888
Q2	0.269	0,002	0.761	0.000	0.078	0.378	0.187	0.033	0.048	0.587	−0.016	0.858	0.233	0.008
Q3	0.037	0.676	0.108	0.223	0.781	0,000	0.354	0.000	−0.053	0.552	0.135	0.125	0.134	0.130
Q4	−0.031	0.728	0.123	0.162	0.153	0.083	0.736	0.000	−0.033	0.709	0.172	0.051	0.049	0.580
Q5	0.214	0.014	0.147	0.096	−0.040	0.649	−0.021	0.813	0.647	0.000	0.146	0.097	0.222	0.011
Q6	0.246	0.005	0.237	0.007	0.178	0.043	0.231	0.008	0.345	0.000	0.749	0.000	0.130	0.142
Q7	0.095	0.284	0.158	0.073	0.153	0.082	0.114	0.196	0.033	0.707	0.003	0.971	0.639	0.000

Table Legend: Rs = Correlation Coefficient; P = value; Q = Question; F = Factor/Choice parameter.

Q1 - What indications do you know for Cannabis-based medicines? - Treatment of depression; Q2 - What indications do you know for Cannabis-based medicines? - Sleep disorders, Q3 - What indications do you know for Cannabis-based medicines? - Cancer Treatment; Q4 - What indications do you know for Cannabis-based medicines? - Treats nausea and vomiting resulting from chemotherapy; Q5 - What indications do you know for Cannabis-based medicines? - Promotes a feeling of well-being; Q6 - What indications do you know for Cannabis-based medicines? - Returns homeostasis (general balance); Q7 - What indications do you know for Cannabis-based medicines? - Chronic pain; F1 - What uses do you know for Cannabis-based Food Supplements? - Treatment of depression; F2 - What uses do you know for Cannabis-based Food Supplements? - Sleep disorders; F3 - What uses do you know for Cannabis-based Food Supplements? - Cancer Treatment; F4 - What uses do you know for Cannabis-based Food Supplements? - Treats nausea and vomiting resulting from chemotherapy; F5 - What uses do you know for Cannabis-based Food Supplements? - Promotes a feeling of well-being; F6 - What uses do you know for Cannabis-based Food Supplements? - Returns homeostasis (general balance); F7 - What uses do you know for Cannabis-based Food Supplements? - Chronic pain.

## 4 Discussion

The results in this study show that the vast majority of respondents are unaware that the indications of medicines cannot be associated with a food supplement, since from the various answer possibilities, descriptive answers of disease-curing properties were selected for the uses of supplements, and these can only be associated with a medication.

A significant correlation was observed between the perception of a relation between medicines with indications for the treatment of depression and cannabis supplements for the same purpose (Rs = 0.603; p = 0.000 < 0.05; n = 130). As for medications with sleep disorder indications, several significant correlations were found relating cannabis supplements for the treatment of depression (Rs = 0.269; p = 0.002 < 0.05; n = 130); sleep disorders (Rs = 0.761; p = 0.000 < 0.05; n = 130) and pain (Rs = 0.233; p = 0.008 < 0.05; n = 130). Concerning medicines with cancer treatment indications, several significant correlations were found with cannabis supplements for the same purpose (Rs = 0.781; p = 0.000 < 0.05; n = 130) and treatment of nausea and vomiting as a result of chemotherapy (Rs = 0.354; p = 0.000 < 0.05; n = 130). A significant correlation was observed between medicines for the treatment of nausea and vomiting as a result of chemotherapy and cannabis supplements for the same purpose: (Rs = 0,736; p = 0,000 < 0,05; n = 130). Concerning medicines with indications for promoting well-being, several significant correlations were found with the perception of cannabis supplements for treating depression (Rs = 0.214; p = 0.014 < 0.05; n = 130); promoting well-being (Rs = 0.647; p = 0.000 < 0.05; n = 130) and treatment of chronic pain (Rs = 0.222; p = 0.011 < 0.05; n = 130).

In the case of medicines with indications to promote homeostasis, several significant correlations were found with cannabis supplements use for the same indication (Rs = 0.749; p = 0.000 < 0.05; n = 130), and for treatment of depression (Rs = 0.246; p = 0.005 < 0.05; n = 130), sleep disorders (Rs = 0.237; p = 0.007 < 0.05; n = 130), cancer (Rs = 0.178; p = 0.043 < 0.05; n = 130), nausea and vomiting as a result of chemotherapy (Rs = 0.231; p = 0.008 < 0.05; n = 130) and promoting well-being (Rs = 0.345; p = 0.000 < 0.05; n = 130). As for medicines with indications for the treatment of chronic pain, it was possible to observe a significant correlation with the perception of cannabis supplements with the same aim (Rs = 0.639; p = 0.000 < 0.05; n = 130).

After carrying out the survey, it was found that the vast majority of respondents (93 %, 164 individuals) were unaware of the characteristics and properties of food supplements in general, which was confirmed by the confusion expressed between the different reasons for using a medicine and a food supplement. It should be noted that 39.20 % state that a supplement has preventive or curative properties, which can only be performed by medicines. Therefore, most participants in this study who adhere to cannabis-based products are unaware that food supplements are foodstuffs intended to complement the normal diet and may only have an indication of nutritional or physiological effects. Similar findings were reported in a study [19] conducted in a Canadian population, where therapeutic effects were attributed to CBD. At the same time, THC was linked with both positive and negative non-therapeutic effects. Another survey conducted in Poland revealed that caregivers of patients with Alzheimer's disease felt healthcare professionals should include CBD oil among treatment options [20]. The need for an increase in health literacy becomes apparent from the results of these studies, and it is important to provide relevant information to consumers.

Among the results obtained, the high degree of satisfaction of respondents who tried products derived from Cannabis stands out, as at level 5 a rate of 30.30 % was obtained, followed by level 4 of 33.30 % and level 3 of 21.20 % (total:84.80 %).

The majority of respondents purchase Cannabis-derived products to combat sleep disorders (48, 50 %) and promote well-being (48.50 %), which in most cases are linked to anxiety [21]. Similar results were obtained in other studies [22,23]. However, in a survey conducted with around 45 000 respondents in the USA and Canada, Cannabis-derived products were mainly used for the management of pain, as well as depression and anxiety [24].

Worthy of note, a significant amount of cannabis food supplements available either in pharmacies or online are based on hemp seed oil (*Cannabis sativa* L. with limited THC content) is characterized by containing a series of fatty acids, namely linoleic acid, α-linolenic acid, and oleic and eicosaenoic acids in smaller quantities [25], known as essential fatty acids, since mammals do not synthesize them and, therefore, must be acquired through the diet as they are necessary to maintain a healthy state. They are necessary for many forms of physiological processes, including the maintenance of cell membrane structure and cardiovascular health, the regulation of metabolic and inflammatory processes through the synthesis of prostaglandins and leukotrienes, as well as the proper regulation of brain development and function [26]. Thus, we subsequently find recommendations associated with supplements containing cannabis oil, for example in online descriptions, such as contributing to the normal functioning of the nervous system, helping to reduce muscle inflammation, improving stress resistance, and physical and mental performance and to overcome fatigue [13]. However, these claims are not proven for the amounts of essential fatty acids present in these products [27].

In the question regarding where knowledge of Cannabis-derived products was obtained, for a significant number (17.70 %) of respondents, it was by suggestion of health professionals, those who are supposed to provide professional advice and monitoring of the consumption of this type of products sought after for health and well-being. Interestingly, in the work by Gurnay et al. [22] targeting a population of anxious users, family, and friends were the prevalent sources influencing decisions to use Cannabis-derived products for anxiety, followed by popular and scientific literature.

Considering the antioxidant and anti-inflammatory properties related to the components of Cannabis seeds, different authors investigated whether food supplementation with products based on Cannabis seeds could have the ability to combat chronic and degenerative diseases risks characterized by inflammation and stress conditions. oxidative, such as cardiovascular and neurodegenerative diseases. So far, most studies have been carried out in animal models and only a few reports have been found in the literature on studies in humans, highlighting the need to investigate the effects of supplementation with Cannabis seeds and their derivatives on human health, in order to understand its potential as an ingredient for foods likely to be associated with nutritional and function claims [28].

Regarding the form of acquisition, the online sales channel stood out (27.3 %) when compared to physical stores (pharmacy, parapharmacy, and herbalist, totaling 21.2 %), with a similar percentage for acquisition in pharmacies and herbalists and a lower in parapharmacies (3 %). Nevertheless, more than half of the respondents (51.50 %) reported that another route was used, neither online nor through regulated stores, nor a defined location, as generally known by society, so it is considered the “Street Market”. If, on the one hand, the issue of regulation could be an obstacle to the acquisition of cannabis-based products since the lack of information can create doubts regarding their nature and accessibility, unregulated access seems to be underlying for more than half of the sample of this study.

Regarding knowledge of the cannabinoids present in these products, 10 % of respondents do not know that THC is absent, and 69.20 % are not aware of the legal percentage that a supplement can contain of this active molecule. This fact highlights the lack of widespread information communicated to consumers has serious effects if this lack of knowledge affects operators and distributors, through inadvertent exposure to high levels of products that may not be controlled after becoming available on the market. The majority of respondents do not know what an acceptable value of THC for human consumption is, which is especially significant considering the case where products are being purchased on an open market, such as online sales.

Given this context, it is possible to hypothesize that if these products become properly regulated or inspected and purchased in places where there are informed and certified health professionals, such as pharmacies or herbalists, the consumer's lack of knowledge about cannabinoids and their legal or acceptable quantities for human consumption becomes less relevant.

It is common sense that consumers of food supplements seem to follow trends, in search of the magic potion, regardless of what they know or don't know, but motivated by what they believe to know.

With instant news on the internet, today's standard consumer still uses a set of search tools that vary greatly between generations, and the impulse buying motivations that already exist seem very different today. With more people browsing for products and services through online platforms, marketers are adjusting their approach depending on what motivations they want to satisfy.

As different generations are challenged online [29], it can also be an issue for regulatory agencies and consumer platforms to advise best practices.

Social media ads targeting older groups can be more product or information-focused, according to what we see on classic search engines. Information aimed at the gray generation is often filled with tips, product descriptions, and written reviews – an indication that their fans dive into content-based research rather than diving in head first.

Conversely, younger consumers make impulse purchases for reasons, such as endorsements from their online community or flexible payment options. And it's worth emphasizing convenience, as that partially explains why they look for innovative products on social apps.

There is also a growing desire for speed throughout the entire purchasing journey, not just at the beginning. Easy or quick online checkout processes (34 %) and “buy” buttons on social media (21 %) are common triggers for impulse buying.

Either way, companies need to construct their online shopping experience around the expectations created by social media searches, enabling a dynamic, fast-paced, and inspiring adventure. Whatever health and/or nutritional benefit-risk is perceived, if it is not communicated, it is not assimilated. But if Authorities assume their role of actively communicating, they must also renew themselves generationally, and become present on social networks, and social apps, or it will not be surprising that consumers increasingly move away from reference knowledge.

Through this value chain, what future will be in store for producers and distributors? They will increasingly respond to the need for seduction, hopefully, with responsibility, well-informed, and understanding that the barriers placed by the Authorities are, in the rule of law, their greatest defense and the guarantee of the protection of public health.

One of the questions that arises here is to understand whether the paradigm of online purchasing and its implications for acquisition by different actors constitutes an unconscious process, or merely the result of the evolution and change in the structure of consumption itself, which follows the attitudes of consumer and their satisfaction. Part of the answer has to do with the type of information that advertising and direct marketing provide to the consumer, in line with the two main techniques used to encourage purchases, as well as to influence the attitudes and opinions of consumers themselves [30].

In this context, Portugal is one of the countries where the influence of the media has the greatest impact on the choices of consumers concerning different products. In the case of Cannabis-based products, this fact, in parallel with the gaps in information and awareness about what constitutes a supplement and medicine, makes it urgent to review the current situation from the perspective of consumer protection, to promote a more conscious and informed choice in the future.

### 4.1 Policy implications

Laws governing recreational use affect the food market and medicinal cannabis use by changing supply and demand, costs, taxation, purity and potentially hazardous contaminants, and potency-related effects.

CBD can be derived from hemp or non-hemp plants, or chemical synthesis. Hemp is defined as any part of the *Cannabis sativa* plant with no more than 0.3 % tetrahydrocannabinol (THC), the psychotropic cannabis main compound. CBD, and cannabinoids in general, are novel foods and cannot be legally included in food or food supplements until a safety assessment has been completed and their use as a novel food has been authorized. Hence, in the European Union (EU), cannabidiol (CBD) products extracted from *Cannabis sativa* L. require pre-marketing authorization under the novel food regulation.

In the UK CBD oil derived from cannabis/hemp plant containing less than 0.2 % THC content is legal except if they claim to be medicine and not a nutritional supplement. Since 2020, CBD food and drink required a Novel Food authorization issued by the UK's Food Standards Agency (FSA). This process involves submitting a dossier of toxicological data for review and approval by the Agency. There are no strict requirements for importing and selling CBD products in the UK provided it is below the detection limit.

For the EU market, data gaps and uncertainties have been addressed by the EFSA's expert Panel on Nutrition, Novel Foods, and Food Allergens (NDA) after 19 applications for CBD as a novel food, with more in the pipeline. They have identified several hazards related to CBD intake and determined that the many data gaps on these health effects need more data before these evaluations can go ahead, however they stressed that they have not concluded that CBD is unsafe as food [31]. There is still insufficient data on the effect of CBD on the liver, gastrointestinal tract, endocrine system, nervous system, and people's psychological well-being. Studies in animals show significant adverse effects, especially in reproduction. It is important to determine if these effects are also seen in humans. In the EU, it's the responsibility of applicants to fill data gaps and EFSA experts are still engaging with them to explain how the additional information can be provided to help address the uncertainties.

On Nov. 21, 2023, the USA Food and Drug Administration (FDA) sent a series of warning letters to companies selling food products containing CBD, though some of those warnings focused on claims that CBD products treat or cure disease. Interestingly, a 2021 survey featured in the USA CBD Consumer Report found that CBD users were primarily hoping for FDA approval and regulation (34.8 %), while others favoured a blend of FDA oversight and industry self-regulation (29.7 %) [32].

In essence, policy implications arise for all European and global markets. For EFSA and the European Parliament it is essential to establish a framework in which individuals can place trust in the precise composition and the risk-to-benefit ratio of emerging foods and dietary/food supplements based on cannabis, CBD and other cannabinoids considered non-psychotropic but with potential medicinal potential. Failure to address the discrepancies and gaps in existing regulations could foster an unregulated parallel market. Such a market runs counter to the principles of a well-regulated economy and raises concerns about the health risks faced by individuals who resort to these products.

Furthermore, it is crucial to highlight a critical aspect: until the completion of safety assessments by EFSA, along with the subsequent inclusion of CBD in the Union List of authorized novel foods, products containing CBD cannot be legally marketed as foods, including dietary supplements.

The use of cannabis worldwide without proper regulation of manufacturing and supply, together with ready access to unregulated, and often illicit, markets of high-concentration products, can result in major societal risks and harms. Organized and transparent regulatory policies, allied with measures to mitigate individual and societal harms should therefore be implemented rapidly.

### 4.2 Strengths and limitations of the study

This study is supported by original data collected by the analysis of the perception of the use of cannabis-based products by consumers. This framework has led to the development of novel knowledge that aims to contribute to improving public health and inform new regulations for the future. A rigorous divulgation of information on cannabis-based products to consumers is an ethical issue that should be a core objective of the pharmaceutical industry and authorities in the health and nutrition fields. The latter could be considered the main limitation of the study because of the communication models of the actual market, the lack of medical knowledge on the correct use of cannabis-based products, and some economic interests that revolve around cannabis.

## 5 Conclusions

Recognizing that the desired biological effects of cannabis products are primarily driven by cannabinoids, specifically THC and CBD, as substantiated by scientific research, it becomes evident that these, alongside other less prevalent cannabinoids, are not authorized for sale in food supplements or related products. This situation raises concerns that currently available cannabis-derived products in the market might either offer a mere placebo effect or conceal cannabinoids in an undisclosed manner, potentially leading to irregular sales.

Considering this context in conjunction with the satisfaction levels reported in this study by users of these cannabis-derived products, expecting potential benefits for well-being and relief from conditions such as Parkinson's disease, epilepsy-related disorders, and muscle relaxation, it becomes increasingly imperative to implement actions. These actions must include better information dissemination for professionals and consumers and imminent regulatory decisions.

It will certainly be interesting in future studies to know whether the consumption of Cannabis-based products authorized as food supplements, containing predominantly unsaturated fatty acids, with potential action on the normal functioning of the heart and contributing to normal blood cholesterol levels, among others, and terpenoids, with antioxidant and other properties, is included in the group consumed for well-being and other desired effects. In light of these considerations, it is clear that the ongoing developments in this sector are substantial, with a burgeoning market segment. It is of paramount importance to undertake measures that will steer this sector in a secure and well-regulated direction, facilitating a conducive environment for laboratories, distributors, and consumers alike. For well-being based on the valorization of knowledge, we propose a formal Knowledge Community collaboration between authorities-industry-academia authorities and industry-academia with citizen involvement for future co-creation of codes of practice.

## CRediT authorship contribution statement

**Alexandre Elias:** Writing – original draft, Investigation. **Catarina Rosado:** Writing – review & editing, Supervision, Data curation. **Maria do Céu Costa:** Writing – review & editing, Supervision, Project administration, Methodology, Conceptualization.

## Ethics and consent declaration

The potential ethical risks of the project to participants were evaluated by peer review and the risks were judged to be low. Review and approval by an ethics committee was not needed, since the study was conducted in compliance with Regulation (EU) 2016/679 of the European Parliament and of the Council of April 27, 2016, as well as Organic Law March 2018 of 5 December, on the Protection of Personal Data and Guarantee of Digital Rights. The ethical principles for medical research involving humans as stated in the Declaration of Helsinki were followed. The questionnaire was conducted anonymously, openly, and was freely accessible, without offering any specific incentives targeting particular adult groups. No personal information was collected from respondents such as name, address, telephone number, or e-mail address. No identifiable information, such as names, addresses, phone numbers, or email addresses, was collected from participants. The purpose of the study was explained to the participants, as well as how their responses would be used at the beginning of the online questionnaire (Appendix 1). All participants were informed that consent to participate in the study and publish their data would be assumed on completion and submission of the study questionnaire.

## Data availability statement

The anonymized data supporting this study's findings are available from the corresponding author upon request.

## Funding

This research was funded by FCT - 10.13039/501100019370Foundation for Science and Technology, I.P. DOI 10.54499/UIDP/04567/2020, DOI 10.54499/UIDB/04567/2020.

## Declaration of competing interest

The authors declare that they have no known competing financial interests or personal relationships that could have appeared to influence the work reported in this paper.

## Figures and Tables

**Table:** 

	Assinalar a resposta
Profissional saúde	
Televisão	
Internet	
Revista	
Outro	

**Table:** 

	Assinalar a resposta
Dores	
Depressão	
Ansiedade	
Fibromialgia	
Distúrbios no sono	
Parkinson	
Alzheimer	
Diabetes	
Cancro	
Outro	

**Table:** 

	Assinalar a resposta
Desconheço a percentagem	
1 %	
2 %	
3 %	
4 %	
5 %	

**Table:** 

	Assinalar a resposta
Dores	
Depressão	
Ansiedade	
Fibromialgia	
Distúrbios no sono	
Parkinson	
Alzheimer	
Diabetes	
Cancro	
Outro	

**Table:** 

	Assinalar a resposta
Loja On-Line	
Farmácia	
Para-Farmácia	
Ervanária	
Grande Superfície	
Outro	

**Table:** 

	Assinalar a resposta
1	
2	
3	
4	
5	

## Appendix 1

ESTUDO DE PERCEÇÃO DE EFICÁCIA DOS UTILIZADORES DE PRODUTOS À BASE DE CANABIDIOL E DO SEU MERCADO.

UNIVERSIDADE LUSÓFONA | LISBOA-PORTUGAL.

O objetivo geral do projeto a ser desenvolvido, é obter dados sobre o consumo e perceção de eficácia de produtos à base de canabidiol, numa amostra de conveniência.
O inquérito será anónimo, sendo usado apenas para o fim deste estudo, e os resultados poderão ser usados em publicações científicas.

Em caso de dúvidas, deverá contactar a equipa de investigação:

Investigador responsável- Maria do Céu Costa: maria.costa@ulusofona.pt.

Muito obrigada por participar desta pesquisa!

Consentimento informado.

Declaro que aceito participar no referido estudo. Entendo que a minha participação é totalmente de forma voluntária e tenho o direito de desistir em qualquer memento, as respostas são anónimas e os resultados desta pesquisa serão tratados apenas para fins de estudo. Li e entendi o consentimento informado.

o Aceito participar no estudo.
o Não aceito participar no estudo.
• *Idade:*

18–30.

31–40.

41–50.

61–70.

70>

• *Género:*

Masculino

Feminino

Não específico.

• *Qual o seu grau académico?*

9° Ano.

12° Ano.

Licenciatura

Mestrado

Doutoramento

• *Conhece os produtos/suplementos à base de Canábis?*

Sim.

Não.

• *Como obteve conhecimento?*

• Quais as indicações que conhece dos produtos de Canábis?

• Conhece a percentagem de THC que se considera seguro tomar num produto de Canábis?

• *Já tomou algum produto derivado de Canábis?*

Sim.

Não.

• *Em caso de toma, qual foi o propósito?*

• Caso tenha tomado, onde adquiriu?

• *Como classifica o resultado obtido, numa escala de 1 a 5, sendo 1 - Nada satisfeito com resultado obtido, e o 5 - Muito satisfeito com o resultado obtido:*
